# An Update on the Correlation Between Neuroimmunomodulation, Photodynamic Therapy (PDT), and Wound Healing: The Role of Mast Cells

**DOI:** 10.3390/biomedicines14020280

**Published:** 2026-01-27

**Authors:** Montserrat Fernandez-Guarino, Luis Alonso-Mtz de Salinas, Jorge Naharro-Rodriguez, Stefano Bacci

**Affiliations:** 1 Dermatology Department, Ramon y Cajal University Hospital, Universidad Alfonso X el Sabio, 28034 Madrid, Spain; drafernandezguarino@gmail.com (M.F.-G.);; 2 Research Group in Dermatology, Photobiology, Light-Based Therapies and Skin Cancer Prevention, Universidad Alfonso X el Sabio, 28034 Madrid, Spain; 3 Research Unit of Histology and Embryology, Department of Biology, 50134 Florence, Italy

**Keywords:** mast cells, neuroimmunomodulation, PDT, wound healing

## Abstract

This review presents some aspects of the complex relationship between neuroimmunomodulation, photodynamic therapy, and wound healing. This relationship is important because photodynamic therapy, currently used to treat chronic wounds, has numerous effects on so-called neuroimmunomodulation, or the influence the nervous system has on immune cells. Consequently, the cellular and molecular mechanisms of wound healing and the alterations of these mechanisms that lead to the formation of chronic wounds are first considered. This view is subsequently broadened to include the effects produced by neuroimmunomodulation throughout the various phases of wound healing and the alterations produced in chronic wounds. Throughout the above, the role of mast cells, the main inflammatory cells that play a key role in wound healing, is highlighted. In this context, mast cells, located in close anatomical and functional proximity to peripheral nerve endings, act as key neuroimmune intermediaries. Upon activation, mast cells release inflammatory mediators that directly influence wound tissue and sensitize nearby nerve fibers. In turn, peripheral nerves release neuropeptides that further modulate immune cell activity, vascular responses, and tissue repair processes. All of this is in turn linked to the clinical evidence that photodynamic therapy, by virtue of the cellular and molecular mechanisms involved, can indeed be considered involved in the healing of chronic wounds.

## 1. Approach

**Figure 1:**
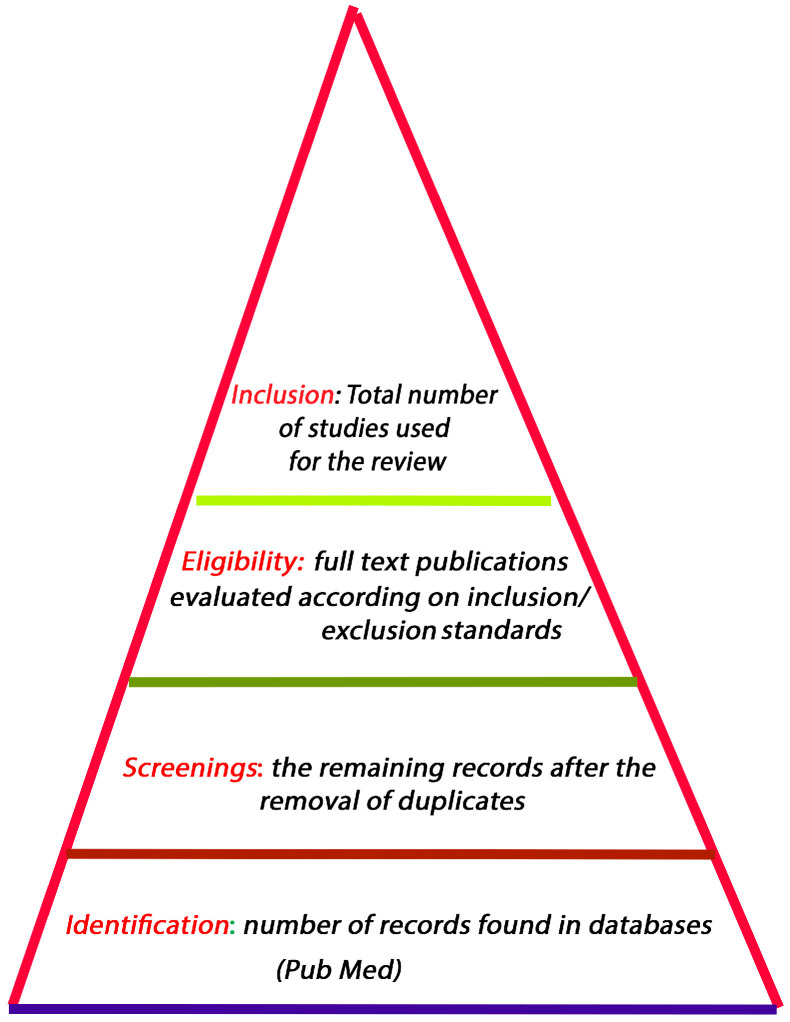
Diagram showing the criteria for selecting the scientific articles in the review.

For this review article, the authors used PubMed as their primary scientific database. Articles were evaluated based on their titles and abstracts. In several cases, the full text, including the conclusions, was also checked (Figure 1).

## 2. Introduction

Challenges associated with the study and management of impaired Wound Healing (WH) are a significant issue in public health, requiring costly and intricate therapies for its control. Millions of people need treatment for chronic wounds (CW), incurring expenses of up to billions of USD. This burden is increasing, mostly due to the rising prevalence of aging, diabetes, and other risk factors among the population. A similar situation is seen in Europe, where it is anticipated that more than 1.5 million individuals may be impacted by CW. As a result, WH is esteemed, shown by the many fellowship programs accessible to various medical experts in this field, including vascular surgeons, nurses, dermatologists, and general practitioners. There is a need for the implementation of modern technologies in WH. Moreover, additional issues arise due to the lack of widespread understanding of the biological mechanisms involved in wound healing; instead, attention is primarily directed towards specialized problems, where, unlike in some fields, few scholars engage in discourse regarding the identified issues [1,2,3,4,5].

## 3. Phases of Wound Healing

### 3.1. Hemostasis

Endothelial cells secrete von Willebrand factor during hemostasis [3,5]. This factor facilitates platelet adhesion, hence initiating the release of mediators. The release of these molecules triggers the creation of a fibrin clot, which obstructs the lesion, stops the bleeding, and prevents further harm. In response to increased calcium ion concentrations, the smooth muscles contract, leading to rapid arterial constriction. This therefore results in a reduction in blood flow via the arteries. These events lead to the formation of vasoactive metabolites, which facilitate vasodilation and the relaxation of arterial vessels. Completing this step needs simply a few minutes [2,3,5].

### 3.2. Inflammation

Mast cells (MC) facilitate vasodilation during the inflammatory phase. This is achieved by the production of histamine or serotonin. In this scenario, neutrophil granulocytes and monocytes, primed to develop into macrophages, engage in a process termed diapedesis. As a result, phagocytosis is activated inside the lesion to eradicate any pathogens or compromised cells that may exist. Leukocytes synthesize cytokines and growth factors to initiate the proliferative phase of the cell cycle. Keratinocytes and several other cell types facilitate this process by secreting cytokines that enhance inflammation. This period often lasts from 0 to 3 days [2,3,5]. Attention should also be directed to the presence of auxiliary molecules throughout the inflammatory phase, including cytokines, matrix proteins, and enzymes. Chemokines are crucial since they mostly attract neutrophils and lymphocytes, which govern the first phases of wound healing [2,3,5].

### 3.3. Proliferation

Fibroblasts have a role in the process of angiogenesis and contribute to the production of granulation tissue during the proliferative phase of the maturation process; additionally, they are responsible for regulating the migration and proliferation of keratinocytes. Throughout this process, macrophages and other cells continue to release growth factors that are involved in the process [2,3,5]. This phase may last anywhere from three to twelve days, depending on the circumstances. Myofibroblasts, which are cells that are positive for Smooth Muscle Actin (SMA) that are produced from fibroblasts, are responsible for driving wound contraction. The formation of collagen and the contraction of the wound are the primary mechanisms that differentiate the maturation or remodeling phase from the remodeling phase. It is the transitions between mesenchymal and endothelial–mesenchymal phenotypes that are governed by a variety of growth factors, which also provide assistance to the remodeling phase. Furthermore, these alterations take place via the beta-signaling pathways of Transforming Growth Factor (TGF) or Notch, both of which lower the amount of cadherin that is produced in vessels [2,3,5]. For the epithelial–mesenchymal transition (EMT) process to take place, beta2AR is an essential molecule that acts as a mediator.

### 3.4. Remodeling/Maturation

Matrix metalloproteinases (MMPs) originating from cells of connective tissues [see 4 for details] and their inhibitors, termed tissue inhibitors of metalloproteinases (TIMPs), are essential in the process of scar formation. The remodeling of granulation tissue is an essential phase in scar formation. The synthesis of the extracellular matrix (ECM) is compromised, resulting in alterations to its composition. Type III collagen precisely replaces type I collagen. Elastin, previously absent, is now present in the granulation tissue. Furthermore, the loss of certain cell types inside the granulation tissue is undoubtedly a critical event in the wound healing process. This study investigates whether dermal fibrocytes derive from other cellular types that develop later in the specified processes or from myofibroblastic forms that gradually lose their unique morphological features [2,3,5].

## 4. Molecular Biomarkers in Wound Healing

The wound healing process is mostly regulated by the intricate interaction of cytokines and growth factors, with any interruption possibly resulting in chronic wounds. Principal pro-inflammatory cytokines, including IL-1beta, TNF-alpha, and IL-6, are essential in the recruitment of inflammatory cells to the site of damage. Macrophages and fibroblasts secrete diverse growth factors such as TGF-beta and Platelet-Derived Growth Factor (PDGF), which are essential for recruiting fibroblasts and promoting the proliferation of epithelial cells. The significance of Vascular Endothelial Growth Factor (VEGF) and Platelet-Derived Growth Factor (PDGF), both released by macrophages, fibroblasts, and keratinocytes, lies in their ability to stimulate endothelial cells and initiate angiogenesis [4,6,7].

Numerous factors affect this healing process, including transcription factors (particularly the E2F family), signaling pathways including Wnt/beta-catenin, STAT3, homeobox genes, and hormonal receptors (androgens, estrogens, glucocorticoids). Significant participants also include Peroxisome Proliferator-Activated Receptors (PPARs), Activator Protein 1 (AP-1), c-Myc, and ETS-Related Gene (ERG) 1, in addition to proteases such as matrix metalloproteinases (MMPs) and diverse enzymes that preserve cellular redox balance. These components engage, illustrating their connection [4,6,7].

The activation of Protein Kinase C (PKC) and Ca^2+^/Calmodulin-Dependent Protein Kinase (CaMK) is essential after the first increase in intracellular Ca^2+^ concentration resulting from injury. This signaling system profoundly affects critical biological processes such as cellular communication, migration, adhesion, and re-epithelialization. Tissue injury also induces Ca^2+^ waves, which activate the RHO family, promote actin polymerization, and augment acto-myosin contractility essential for maintaining stroma and structural integrity [4,6,7]. GTPases facilitate actin polymerization and acto-myosin contractility.

The release of calcium initiates many signaling pathways, including c-Jun N-Terminal Kinase (JNK) and Mitogen-Activated Protein Kinase (MAPK), which then activate transcription factors and enhance the expression of genes associated with stress responses, particularly those related to cytoskeletal dynamics. Finally, purinergic receptors are essential in wound healing by modulating ATP release and activation, since neighboring epithelial cells sense DNA damage via P2Y receptors, resulting in increased intracellular calcium activation and matrix metalloproteinase activity. This cascaded reaction facilitates the release of growth factors, such as EGF, which are crucial for the wound healing process [4,6,7].

Calcium ions have a crucial function as signaling molecules in the repair mechanisms of skin cells, such as keratinocytes and fibroblasts, affecting inflammation, proliferation, and remodeling. Inadequate calcium impedes these activities, resulting in delayed wound healing. Calcium influx initiates essential cellular functions, facilitating the migration and proliferation required for wound healing. Moreover, calcium gradients are essential for keratinocyte development, which establishes the skin’s protective barrier. It also affects fibroblast activity, hence altering collagen deposition, which is essential for tissue healing [8].

## 5. Genetic Activation in Wound Healing

The functioning of many genes encoding specific molecules (cytokines, chemokines, and growth factors) delineates the characteristics of the several phases of wound healing and their interconnections. The genes *Tyrosinase* (*TYR*), *Tyrosinase-Related Protein 1* (*TYRP1*), and *Dopachrome Tautomerase* (*DCT*) exemplify hub genes involved in melanin synthesis. During the inflammatory and proliferative stages, eighty-five differentially expressed genes (DEGs) and one hundred sixty-four downregulated proteins were identified. Three hub genes are implicated in the P53 signaling pathway and the cell cycle. The genes are designated as *Cyclin B1* (*CCNB1*), *Checkpoint Kinase 1* (*CHEK1*), and *Cyclin Dependent Kinase 1* (*CDK1*). During the remodeling phase, a total of 121 differentially expressed genes and 49 weakly expressed genes were identified. A correlation exists among the hub genes for *Collagen Alpha Chain 1* (COL4A1), *Collagen Type 4 Alpha Chain 2* (*COL4A2*), and *Collagen Type 6 Alpha Chain 1* (*COL6A1*) concerning protein digestion and absorption, along with their interaction with the extracellular matrix receptor [4,6,7]. Furthermore, it is crucial to acknowledge that, in recent decades, scientific research has focused on the impact of individual cytokines on certain characteristics of WH across various experimental contexts. Recent research have identified critical genes implicated in the links between *Interleukin* (IL)17 signaling pathways and other receptors, including *IL1Beta*, *IL6*, or chemokines as *CCL4*, *CXCL1*, *CXCL2*, *CXCL3*, *CXCL5*, *CXCL6*, and *CXCL10*. *IL6* and *IL1beta* are crucial for enhancing keratinocyte motility and facilitating epidermal healing. Both processes are vital for the skin. Finally, it is crucial to note that recent research has shown that the decreased production of *CXCL1* and *CXCL5*, which serve as chemoattractants for neutrophils, reduces the quantity of white blood cells in mice [4,6,7].

Pro-inflammatory genes produced during the first phases of damage activate molecules such as *TNF* (*Tumor Necrosis Factor*) alpha, *Interferon* (IFN) gamma, and *TGF-beta*. The gene profile comprises genes that encode substances like *VEGF*, *PDGF*, *Fibroblast Growth Factor* (FGF)2, and *MMP*. These chemicals may increase fibroblast and keratinocyte proliferation, promote angiogenesis, and facilitate epithelial tissue regeneration throughout the wound healing process. To facilitate collagen synthesis by fibroblasts and the degradation of extracellular matrix during tissue resorption, the genes responsible for *TGF-beta1* and *MMP* expression are upregulated during the remodeling phase. Any alteration in gene expression may influence the healing process. This transition may result in the release of chemicals including cytokines, chemokines, and growth factors, potentially culminating in the formation of chronic lesions [4,6,7].

Moreover, epigenetic pathways have a role in wound healing, but the molecular processes remain incompletely elucidated. A diverse array of cases has been collected regarding these procedures, facilitating the identification of several microRNAs.

These microRNAs regulate inflammatory responses, ECM synthesis, cellular proliferation, and intercellular communication during wound healing processes [4,6,7].

The proteolytic breakdown of fibronectin exemplifies post-translational processes, facilitating cell proliferation and migration during wound healing.

The WH process is characterized by a controlled quality that varies across individuals. Certain mouse strains, such as MRL/MpJ-Faslpr (MRLF), have been shown to heal a 2 mm diameter ear-punched hole within thirty days. Conversely, other mouse strains, including C57BL/6 and SJLJ, exhibit healing rates of forty percent and twenty-five percent, respectively, during the same duration [4,6,7].

## 6. Dysfunction of the Cellular Mechanisms Associated with Wound Healing 

### Chronic Wounds

If the phases of wound healing are not completed within six to eight weeks, the wound is classified as chronic, and its treatment is quite costly [1,2]. The creation of biofilm often impedes wound healing, facilitating the transition from acute to chronic wounds. This is true despite the existence of many forms of chronic wounds. A significant characteristic that differentiates wound microbial communities from other community types is the presence of a diverse array of bacterial species at the infection site [9]. Changes in MMP secretions, rather than acute lesions, are the principal mechanism by which CW sustain the inflammatory phase [10]. The cellular infiltration comprises a substantial quantity of cells that contribute to the pronounced inflammatory response seen in CW. Neutrophils are present in elevated quantities in chronic wounds, and they are accountable for the secretion of substantial quantities of metalloproteinases. These enzymes not only compromise the connective tissue matrix and elastase but also deactivate critical elements necessary for wound healing. These factors include PDGF and TGF-beta. Keratinocytes secrete a diverse array of signaling chemicals. Nonetheless, the extent to which these mechanisms contribute to CW remains unclear. Moreover, the expression of genes linked to diminished proliferative activity in keratinocytes inside chronic wounds elucidates the increased proliferation of the epidermis next to the ulcer margins. Moreover, fibroblasts do not demonstrate any migratory responses when stimulated by TGF-beta [9,10,11]. Chronic wounds have increased concentrations of inflammatory mediators, including cytokines such as IL1β, TNFα, and IL6, which perpetuate a sustained inflammatory condition and recruit more inflammatory cells. Chemokines (e.g., CXCL8, CXCL1, CXCL2) are produced to promote the recruitment of inflammatory cells; nevertheless, in chronic wounds, their sustained presence fosters a prolonged inflammatory milieu. The inflammasome protein complex, which activates and releases IL-1β, is associated with the formation of chronic wounds and ensuing inflammation. Concerning proteolytic enzymes and growth factors, CWs exhibit elevated levels of MMPs that excessively degrade the extracellular matrix, alongside diminished levels of critical growth factors such as PDGF, TGFβ, FGF, and VEGF, all of which are essential for cell migration, proliferation, and angiogenesis [9,10,11]. Concerning further molecular factors, reactive oxygen species (ROS) are crucial for antibacterial activity and signaling in normal healing; however, imbalances in ROS can lead to chronic wound pathogenesis. Conversely, dysregulation of pathways such as the Wnt pathway, marked by increased nuclear β-catenin and c-myc, may result in abnormal proliferation and differentiation of epidermal cells at the wound margin [9,10,11,12]. It has been shown that levels of TGF-betaR and the downstream elements of the TGF-betaR signaling cascade have diminished [9,10,11,12]. Chronic wounds are associated with certain intracellular and cellular/ECM receptor types and signaling pathways. Integrins are essential cell-surface receptors facilitating cell adhesion and migration, with epidermal keratinocytes expressing several integrins that interact with ECM ligands in the provisional wound ECM. These receptors affect interactions between epidermal and dermal cell matrices, cellular motility, cell phenotypic, and the healing process. Chronic wounds exhibit alterations in receptors, notably a reduction in integrin α5β1 receptors, which impairs fibronectin integration and keratinocyte migration. This stimulates TGF β, heightening vulnerability to ulceration and fibrosis, and culminates in an accumulation of senescent cells, resulting in a degradative ECM, compromised wound bed, and corrosive chronic wound fluid. Modulating integrin receptors via different compounds may affect wound healing, enhancing intrinsic repair mechanisms and the production of growth factors and extracellular matrix proteins, perhaps offering a more economical alternative to conventional extrinsic supplementation of these agents [13].

The dynamic reciprocity of the wound microenvironment, characterized by continual bidirectional interactions between cells and their surrounding milieu, is crucial. Cell–extracellular matrix interactions not only direct and regulate cellular morphology but also influence cellular differentiation, migration, proliferation, and survival during tissue development, such as embryogenesis and angiogenesis, as well as during pathological processes including cancer, diabetes, hypertension, and chronic wound healing [5,14].

However, research on wound healing indicates that conventional descriptive models of the four stages (hemostasis, inflammation, proliferation, and remodeling) often do not adequately represent the intricacies of non-linear and chronic healing processes. Contemporary research challenges the inflexible, linear phase model, highlighting that these processes are ongoing and significantly interrelated. The disparity in biomarker translation is a pressing issue, since there is an absence of economical, point-of-care diagnostic instruments for molecular biomarkers. Conventional animal models face growing criticism for their inability to correctly replicate human skin architecture and immunological responses, resulting in a “translational gap” where laboratory results do not translate to human treatment trials. Anticipated developments should be included with the incorporation of artificial intelligence (AI) for the analysis of wound imagery and biomarker data, intelligent and bioactive therapies, customized wound care, and comprehensive integration. These developments seek to overcome the constraints of conventional descriptive models and enhance comprehension of wound healing mechanisms.

## 7. Neuroimmunomodulation During Wound Healing

Neuroimmunology is a burgeoning discipline that examines the intricate relationships between immunological and neurological systems, which profoundly affect health outcomes, behavior, and reactions to environmental stimuli. This research focuses on neuroimmunomodulation, emphasizing the communication pathways that influence both immune responses and brain processes beyond simple linkage. The communication mechanisms include channels via the endocrine system, sympathetic and parasympathetic systems (including cholinergic and vagal pathways), sensory systems, and meningeal lymphatics, signifying a dynamic interaction in immune control facilitated by neurotransmitter receptors and sympathetic fibers associated with lymph nodes.

Recent studies indicate the substantial influence of cytokines on host behavior, even in the absence of infection, suggesting an evolved capacity to improve survival by proactively adapting to environmental stressors. The parallels between the neurological and immune systems highlight their functions as sensors of internal and external stimuli, using receptors like T-cell receptors in lymphocytes and ligand-gated channels in sensory neurons to enable rapid reactions to threats. Both systems exhibit adaptability to various environmental situations and collaborate in signaling pathways, resulting in behavioral modifications and providing insights into their combined functions in health and illness management. This convergence highlights the immune system’s potential as a crucial participant in neuromodulation, modifying its activities in response to diverse inputs for the host’s advantage [15,16,17].

This field has elucidated mechanisms such as the cholinergic anti-inflammatory pathway, where vagal nerve stimulation can suppress systemic inflammation, and the role of neurotransmitters and neuropeptides in regulating immune cell function. These interactions are relevant in pain management, neurodegenerative diseases, and psychiatric disorders, and are increasingly being targeted by pharmacological and bioelectronic therapies to restore neuroimmune homeostasis [15].

In addition, the process of ulcer healing reveals a tight association that is comparable to another. It has been proven via experiments that neurogenic stimuli have a significant impact on the process of wound regeneration that occurs after an injury. It has also been shown via research that delayed wound regeneration occurs in animal models after the surgical removal of cutaneous nerves [16,17].

### 7.1. Inflammatory Phase

During the inflammatory phase of wound healing, neuropeptides such as Substance P are produced from the cutaneous innervation. Substance P augments microvascular permeability and vasodilation by facilitating nitric oxide release and affecting endothelial cells. It further enhances adhesion molecules, facilitates monocyte chemotaxis, and activates inflammatory cell activity. SP modulates the production and secretion of pro-inflammatory cytokines such as interleukins, TGFα, and TNFα. Neutral endopeptidase (NEP) antagonizes the effects of Substance P (SP) and competes with the neurokinin-1 receptor (NK-1R), hence altering inflammatory signals in the context of wound healing. Neurokinin A (NKA) is a bioactive tachykinin that is released into the skin after an injury. It stimulates cutaneous target cells, such as keratinocytes and dermal endothelial cells, mostly via the neurokinin-2 receptor (NK-2R), hence playing a role in the modulation of skin inflammation during WH [18,19,20,21,22].

Sensory cutaneous neurons possess corticotropin-releasing hormone (CRH), as shown by immunohistochemical investigations. Corticotropin-releasing hormone (CRH) triggers the degranulation of cutaneous MC and functions as a pro-inflammatory mediator, hence enhancing vascular permeability and the secretion of pro-inflammatory cytokines. CRH has been shown to promote angiogenesis in the epidermis [3,23,24].

Calcitonin gene-related peptide (CGRP) functions as a vasodilator, facilitating angiogenesis and enhancing plasma extravasation. It amplifies the inflammatory response of other mediators, particularly Substance P. Activin, a member of the TGF-β superfamily, enhances CGRP production in sensory neurons and elevates post-injury, highlighting its regulatory role in wound healing [3,23,24].

Nerve growth factor (NGF) contributes to the inflammatory phase of wound healing by enhancing the release of CGRP from peripheral nerve terminals into surrounding tissue [3,23,24].

Neuropeptide Y (NPY) and CGRP have shown both pro-inflammatory and anti-inflammatory characteristics in murine models. In recent years, the roster of mediators participating in wound healing has broadened to include Nitric Oxide (NO), an extracellular chemical messenger [3,23,24].

Nitric Oxide Synthase (NOS) catalyzes the synthesis of nitric oxide, a process augmented by inflammatory mediators, apoptotic debris, or bacterial antigens. Inducible nitric oxide synthase (iNOS) is proposed to contribute to the inflammatory phase of wound healing by facilitating vasodilation and enhancing antibacterial activity [25].

### 7.2. Proliferative Phase

SP induces DNA synthesis, leading to significant proliferative effects on fibroblasts, keratinocytes, and endothelial cells. It further promotes angiogenesis, maybe via the mediation of nitric oxide (NO). SP is crucial for the remodeling of granulation tissue by facilitating the proliferation and migration of dermal fibroblasts and augmenting the production of epidermal growth factor and its corresponding receptor [18,19,20,21,22,23].

Polypeptide neurotrophins, including nerve growth factor (NGF), are found in neurons of both the central and peripheral nervous systems, as well as in many cell types such as fibroblasts, epithelial cells, keratinocytes, and immune cells. NGF is essential for the survival, functionality, and maturation of sensory and autonomic neurons. Moreover, NGF has anti-inflammatory effects. NGF is believed to promote the proliferation of local immature cells in lesions, boost angiogenesis, and accelerate neurite outgrowth. In animal studies and a human case study, NGF has shown the capacity to promote epithelial repair and angiogenesis. Neurokinin A induces the secretion of NGF in the epidermis [18,19,20,21,22,23].

Other neuropeptides recognized for their role in the proliferative phase are GRP, CGRP, galanin, vasoactive intestinal peptide (VIP), and pituitary adenylate cyclase-activating peptide (PACAP) [18,19,20,21,22,23].

CGRP is widely disseminated throughout the central and peripheral neural systems. Although its effectiveness in enhancing wound healing remains ambiguous, several studies suggest it may promote angiogenesis, keratinocyte proliferation, and cellular migration. CGRP promotes keratinocyte proliferation and migration [3,23,24].

Galanin is a peptide secreted by afferent neurons that has anti-proliferative properties in tissues. It transmits signals via G-protein coupled receptors. Conversely, an in vitro investigation shown that galanin induced the overexpression of NGF [3,23,24].

VIP has shown its function as a growth factor for keratinocyte proliferation and in the regulation of their migration. Moreover, VIP stimulates mast cells to produce histamine, leading to vasodilation. VIP may promote the reinnervation of injured tissue, as it has been shown to expedite the regeneration of the sciatic nerve in rats after transection. Substance P, calcitonin gene-related peptide, and vasoactive intestinal peptide regulate matrix metalloproteinase activities and affect the synthesis of collagen-1 and collagen-3 during cutaneous wound healing [3,23,24].

PACAP is situated inside sensory cutaneous nerves. It is a powerful vasodilator and part of the vasoactive intestinal peptide family. C-fibers are believed to release PACAP following neuronal activation, leading to extravasation and vasodilation. PACAP promotes cutaneous inflammation by enhancing the proliferation of human keratinocytes and inducing histamine production from MC [26,27].

### 7.3. Remodeling Phase

The remodeling phase of cutaneous wound healing entails the regeneration of sensory nerve fibers inside the restored epidermis and dermis [18,19,20,21,22].

Substance P stimulates the synthesis of nerve growth factor by human dermal microvascular endothelial cells, which is essential for nerve fiber regeneration after cutaneous damage [18,19,20,21,22]. NGF has been suggested to accelerate tissue remodeling [18,19,20,21,22].

Sensory and sympathetic nerves produce neurotrophin-3 (NT3), which is a crucial neurotrophic growth factor for nerve development and maintenance.

Brain-derived neurotrophic factor (BDNF) is essential for the survival and functional development of sensory neurons. Keratinocytes, fibroblasts, and myofibroblasts produce BDNF and its receptors, promoting their proliferation and differentiation. Substance P, Calcitonin Gene-Related Peptide, and Vasoactive Intestinal Peptide modulate Matrix Metalloproteinase activity and the production of collagen-1 and collagen-3 in dermal wound healing.

The role of neuropeptides and cutaneous innervation during this period is inadequately understood [3,23,24].

### 7.4. Neuroimmunomodulation in Chronic Wounds

The interrelations between the immune system and the neurological system are substantial in their ability to regulate WH processes [15,16]. Recent studies indicate that the transmission of neurotransmitters, including Substance P (SP), protein gene product 9.5 (PGP 9.5), nitric oxide (NO), nerve growth factor (NGF), neurokinin A (NKA), neuropeptide Y (NPY), calcitonin gene-related peptide (CGRP), and vasoactive intestinal peptide (VIP), is crucial in chronic wounds. This mechanism is enabled by interactions between MC and nerve cells, resulting in the secretion of ECM by fibroblasts, an elevation in TGFbeta levels, and the activation of invading cells [27,28].

## 8. Photodynamic Therapy

**Figure 2:**
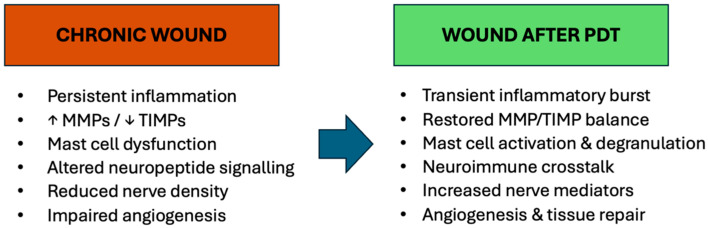
Effect of photodynamic therapy on the chronic wound microenvironment. Schematic comparison between untreated chronic wounds and chronic wounds after PDT. PDT induces a transient inflammatory response and neuroimmune activation, characterized by mast cell degranulation, neuropeptide release, and restoration of immune–neural communication, ultimately promoting angiogenesis and tissue repair.

Photodynamic therapy (PDT) is well documented and recognized in the medical domain for the treatment of both oncological and non-oncological disorders [1,29,30]. In dermatology, applications encompass oncological conditions including basal cell carcinoma, squamous cell carcinoma, actinic and non-oncologic keratoses, alongside bacterial, fungal, viral, immunological, or inflammatory infections, in addition to the management of chronic wounds and cosmetological procedures for photorejuvenation [31]. PDT depends on the cytotoxic properties of certain reactive oxygen species, particularly unstable oxygen molecules that rapidly engage with cellular constituents. A buildup of reactive oxygen species may cause damage to DNA, RNA, and proteins, possibly resulting in cell death. This encompasses singlet oxygen, superoxide anions, and hydroxyl radicals generated by the transfer of energy and/or electrons from the photoexcited oxygen sensitizer. Three primary mechanisms support the effectiveness of PDT: (1) direct cytotoxicity or inflammation of neoplastic cells, (2) disruption of tumor vasculature, and (3) an immunological response marked by leukocyte activation and the release of interleukins, cytokines, growth factors, complement components, acute phase proteins, and other immunomodulators [1,31,32]. Recent research in wound healing illustrates the efficacy of PDT due to its antibacterial qualities, its ability to target biofilms, and its influence on the extracellular matrix via the activation of MMPs, hence promoting modifications in collagen for tissue regeneration. Moreover, PDT induces cellular modifications, which is a characteristic seen during tissue repair mechanisms [1,31,32] (Figure 2).

PDT is a treatment that utilizes a photosensitizer (administered either topically or systemically), light (which interacts with the photosensitizing agent), and oxygen to induce selective cell death through necrosis or apoptosis in cells that are “atypically” sensitized, where the photosensitizer or its precursor accumulates selectively, either topically or intravenously.

In summary, the photodynamic effect, driven by photophysical, photochemical, and photobiological mechanisms, is regulated by the generation of ROS, and is contingent upon the intracellular interactions between the photosensitizer, light, and oxygen [1,31,32]. The topical use of Aminolevulinic Acid (ALA) a natural precursor of proto-porphyrin IX, which subsequently acts as a precursor for the heme group and a powerful photosensitizing agent, represents a notable improvement in dermatology owing to its excellent skin absorbability [1,31,32]. At the cellular level, the pro-drug, upon conversion to protoporphyrin IX, induces the production of reactive oxygen species, resulting in the death of target cells. The presence of ROS near cellular and subcellular membranes, especially the mitochondrial ridges, promotes the release of cytochrome C, triggering the caspase cascade, which ultimately leads to intrinsic apoptosis. The effect is exacerbated by the deterioration of small arteries via a photodynamic process and the onset of an inflammatory response [1,31,32]. The concentration of 5-ALA generally fluctuates according on the treatment modality, with a systemic range of 2–40% and a topical range of 30–50 mg/cm^2^. It is often given for less than 4 h, with peak accumulation between 3 and 8 h [1,31,32].

### 8.1. Biomolecular Impact of PDT

PDT has been examined for its efficacy in diminishing certain bacteria that generate ROS and in preventing the development of resistance to traditional antibiotics within the framework of constructed wetlands. Furthermore, the decrease in MMP activity and the restoration of collagen must be considered in the context of PDT-induced tissue regeneration. Nonetheless, the use of PDT as an adjunct to CW in clinical settings remains uncommon owing to the limited number of published studies and the need for numerous repeated sessions with the currently available light sources and photosensitizers [1,4,32].

### 8.2. Response of Cellular Infiltrate

Previous study indicates that PDT may induce a transient inflammatory response mostly associated with immune system activation. The observation that PDT induces the diversification of new fibroblasts (effector cells) [33] and enhances the cellular interactions between these cell types and FGFpositive, as well as TNFalpha, MC cells in their granules corroborates the prior assertion [33].

Consequently, the notion that MC could transmit signals that initiate the recruitment and differentiation of additional fibroblasts post-therapy seems reasonable [1,33,34]. The elevation in the degranulation index and the quantity of these cell types subsequent to the treatment further substantiates this theory. The increased MC may result from the differentiation of existing precursors inside the tissue or the inflow of precursors that eventually develop into these cells. The subpapillary plexus seems to be a preferred site for mast cell aggregation and cellular infiltration after treatment [1,33,34]. Mast cell heterogeneity denotes the presence of many subtypes, such as mucosal mast cells (MCT) and connective tissue mast cells (MCTC), which differ in their protease composition and anatomical distribution. Mast cells of the type MCT are often situated in the gastrointestinal tract and lungs, possessing tryptase, while MCTC are distributed in the dermis and peritoneal cavity, including both tryptase and chymase. This distinction is crucial as it influences the efficacy of therapies like Photodynamic Therapy (PDT). The specificity of PDT’s effects on certain mast cell subtypes may influence many biological responses, including inflammatory reactions, the production of mediators such as histamine and cytokines, and interactions with immune cells. Recognizing the variety among mast cell populations facilitates focused discussions on the processes and effectiveness of therapies such as PDT in particular illness situations, resulting in more precise and successful treatment options [1,34]. The immune system’s activation is also facilitated by the pronounced production of TNFalpha and TGFbeta by MC after PDT. TNFalpha is crucial for the development of certain dendritic cell types, such as plasmacytoid cells that engage with regulatory T lymphocytes. MC also express TGFbeta, which is crucial for macrophage differentiation [33]. The observed decrease in lesion volume post-treatment is undoubtedly linked to the activation of TGF-beta [33]. Indeed, TGFbeta seems to influence the epithelial–mesenchymal transition throughout the different phases of ulcer healing. This shift enables keratinocytes to migrate from the periphery to the wound bed. This cytokine may trigger the differentiation of myofibroblasts, which is a crucial stage in scar remodeling [34].

PDT has been shown to significantly influence neutrophil activation, contributing to the subsequent increase in pro-inflammatory cytokines post-therapy, as indicated by data from previous investigations. During the acute phase of inflammation resolution and the following restoration of tissue homeostasis, the production of lipid mediators occurs simultaneously. These mediators are associated with anti-inflammatory and immunomodulatory properties. Examples of these qualities include the inhibition of leukocyte chemotaxis, the obstruction of TNFalpha and IL6 production, and the resultant increase in IL10 expression [1,34].

PDT is anticipated to exhibit both immunostimulatory and immunosuppressive effects, which are likely to be the determining factor in the kind of cell death induced. Consequently, it is reasonable to conclude that PDT substantially influences the immune system.

These immunological changes include macrophage polarization towards the reparative M2 phenotype, increased secretion of transforming growth factor beta 1 (TGF-β1), and promotion of epithelial–mesenchymal transition, enhancing wound closure and tissue regeneration [35].

### 8.3. Neuroimmunomodulatory Effects of PDT in Chronic Wounds

**Figure 3:**
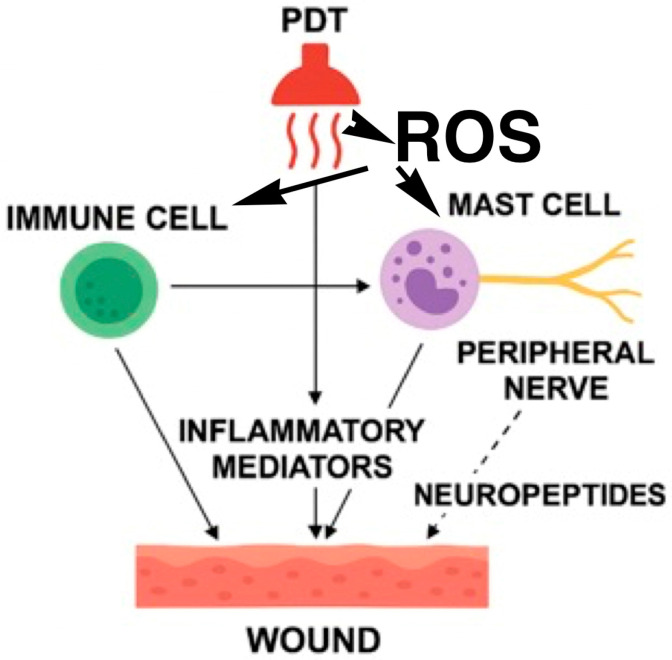
Photodynamic therapy as a neuroimmunomodulatory trigger in wound healing.

There is robust evidence supporting the connection that neuroimmunomodulation, wound healing, and photodynamic therapy are interconnected through their shared reliance on immune system modulation, with photodynamic therapy representing a clinically relevant strategy to harness immune responses for improved wound repair. PDT for chronic wounds has shown a rise in the density of neuronal populations in the dermis, which are part of the autonomic nervous system and contain the characteristic nerve mediators associated with chronic wounds. The percentage of mast cells that can secrete and store NGF, VIP, and iNOs compounds rises after a single irradiation, along with the previously noted increase in the mast cell degranulation index [3,23,24,25]. A single session of ALA-PDT has been shown to induce acute mast cell degranulation accompanied by a peripheral neuropeptide response, indicating that PDT activates not only immune effector cells but also cutaneous nerve terminals [26,27]. Mast cells are strategically localized at the neurovascular interface and act as key intermediaries between the nervous and immune systems; their rapid activation releases histamine, proteases, cytokines, and growth factors that promote neurogenic inflammation, while neuropeptides released from sensory fibers reciprocally regulate mast-cell behavior [17,26,27]. In parallel, increasing evidence highlights the iNOS/nitric oxide (NO) axis as a molecular link between immune activation, vascular regulation, antimicrobial defense, and neural signaling following PDT. Upregulation of iNOS expression and NO production after PDT has been reported in chronic venous ulcers, suggesting a mechanism through which immune-derived mediators influence neurovascular tone and tissue remodeling [25]. Together, these findings support the interpretation of PDT as a context-dependent neuroimmunomodulatory intervention, capable of restoring the coordinated immune–neural dialog required for progression from chronic inflammation to effective wound repair [25,27,28]. In particular photodynamic therapy induces localized oxidative stress, resulting in the release of inflammatory mediators from immune and mast cells. Mast cells are located in close anatomical and functional proximity to peripheral nerve terminals, making them crucial neuroimmune mediators. Upon activation, mast cells release inflammatory mediators that directly affect the injured tissue and sensitize nearby nerve fibers. This activation establishes a feedback loop, whereby peripheral nerves subsequently produce neuropeptides that further regulate immune cell activity, affect vascular responses, and promote tissue healing mechanisms. The interaction among these factors highlights the importance of neuroimmune interactions and inflammatory responses in wound healing (Figure 3).

Schematic representation of the interactions between photodynamic therapy (PDT), immune cells (neutrophils, macrophages, and dendritic cells), mast cells, and peripheral nerves within the wound microenvironment. PDT induces local oxidative stress, leading to the release of inflammatory mediators from immune cells and mast cells. Mast cells, located in close anatomical and functional proximity to peripheral nerve endings, act as key neuroimmune intermediaries. Upon activation, mast cells release inflammatory mediators that directly influence wound tissue and sensitize nearby nerve fibers. In turn, peripheral nerves release neuropeptides that further modulate immune cell activity, vascular responses, and tissue repair processes.

### 8.4. Clinical Evidence of PDT in Chronic Wounds

Clinically, PDT is particularly beneficial in wounds complicated by biofilm-forming bacteria, multidrug-resistant pathogens, and persistent inflammatory responses, where conventional treatment options often prove inadequate or resistant [36]. Due to PDT additional photobiomodulatory properties, PDT shows therapeutic potential in patients suffering from chronic, infected, or non-healing wounds, such as diabetic foot ulcers, burn injuries, and refractory skin scaring, especially in cases where immune dysfunction or microbial resistance limits the efficacy of standard therapies [37]. In dermatological practice, PDT is routinely applied in the treatment of diabetic wounds, burns, and chronic ulcers, frequently in combination with immunomodulatory agents to augment immune-mediated repair mechanisms [38]. While current evidence supports the immunoregulatory and neuroimmune modulatory effects of PDT, further large-scale, controlled clinical trials are essential to optimize treatment protocols and elucidate long-term therapeutic outcomes across diverse patient populations.

Emerging studies have shown that PDT can significantly accelerate wound closure, eliminate microbial biofilms, including methicillin-resistant *Staphylococcus aureus* (MRSA), and promote both angiogenesis and re-epithelialization in preclinical models and early-phase clinical investigations [39]. Thus, offers several advantages over conventional treatments for chronic wounds, including broad-spectrum antimicrobial efficacy, minimal invasiveness, reduced risk of antibiotic resistance, enhanced healing rates, improved scar quality, and stimulation of local immune responses [37]. Moreover, PDT can be effectively combined with advanced wound dressings, nanotechnologies, and adjunct therapies to address complex wound environments characterized by hypoxia, impaired perfusion, or biofilm formation [40].

However, a key limitation of PDT lies in the restricted penetration of light into biological tissues, as well as the difficulty in ensuring precise delivery of the photosensitizing agent. Typically, light can only penetrate a few millimeters into tissue, which limits the therapeutic efficacy of PDT in deeper or more extensive wounds and may necessitate the use of advanced light delivery methods, such as interstitial illumination techniques [41].

Furthermore, sequential therapeutic strategies that combine PDT with anti-inflammatory agents and oxygen-releasing systems have demonstrated improved modulation of the wound microenvironment, thereby promoting more effective and sustained healing outcomes [42,43].

While current evidence supports the immunoregulatory and neuroimmune effects of photodynamic therapy (PDT), further large-scale, controlled clinical trials are needed to optimize treatment protocols and clarify long-term outcomes across varied patient populations. PDT shows particular promise in chronic wound management through its antimicrobial action, modulation of inflammation, and tissue regeneration. Emerging combination strategies—such as the inclusion of neuroimmune agents and advanced delivery systems—may further enhance its therapeutic potential. However, challenges including limited tissue penetration, ROS-associated risks, and the need for standardized protocols must be addressed to ensure broader clinical adoption.

## 9. Conclusions and Prospective of Research

Chronic skin lesions lasting six to eight weeks demonstrate inflammatory processes that hinder normal healing, affected by many comorbidities, especially in those over 65 years of age. Chronic ulcers have elevated protease activity and an abundance of pro-inflammatory cytokines, obstructing tissue healing. Although chronic and acute wound healing pathways are analogous, chronic wounds exhibit dysregulated production of matrix metalloproteinases (MMPs), which extends inflammation as neutrophils release MMPs, therefore compromising the connective tissue matrix.

Infections exacerbate healing by promoting biofilm formation and elevating MMP levels. Photodynamic treatment (PDT) increases neuronal density in the dermis and regulates immunological responses, especially in mast cells, hence facilitating wound healing in individuals with chronic inflammation or compromised immune systems.

In this context, a thorough examination using advanced imaging modalities and high-throughput screening of key biomarkers and the molecular relationships between neuropeptides, immune cells, and signaling pathways could facilitate understanding the mechanisms related to photodynamic therapy.

The use of various animal models with different experimental modalities combined with further clinical studies (including personalized light therapies) could certainly improve our understanding of the subtle cellular and molecular mechanisms of photodynamic therapy [1,4,9,11,26,27,38].

## Data Availability

No new data were created or analyzed in this study. Data sharing is not applicable to this article.

## Abbreviations

The following abbreviations are used in this manuscript:

WH	Wound Healing
CW	Chronic Wounds
MC	Mast cells
SMA	Smooth Muscle Actin
TGF	Transforming Growth Factor
EMT	Epithelial–Mesenchymal Transition
MMPs	Matrix metalloproteinases
TIMPs	Tissue Inhibitors of Metalloproteinases
ECM	Extracellular Matrix
PDGF	Platelet-Derived Growth Factor
FGF	Fibroblast growth factor
KGF	Keratinocyte Growth Factor
EGF	Epithelial Growth Factor
HGF	Hepatocyte Growth Factor
IGF	Insulin-like Growth Factor
VEGF	Vascular Endothelial Growth Factor
STAT	Signal Transducer and Activator of Transcription
PPAR	Peroxisome Proliferator-Activated Receptors
AP1	Activator Protein 1
ERG	ETS-related Gene
PKC	Protein Kinase C
CaMK	Ca^2+^/Calmodulin-Dependent Protein Kinase
ROS	Reactive Oxygen Species
JNK	c-Jun N-Terminal Kinase
MAPK	Mitogen-Activated Protein Kinase
TYR	Tyrosinases
TYRP1	Tyrosinase-Related Protein 1
DCT	Dopachrome Tautomerase
DEGs	Differentially Expressed Genes
CCNB1	Cyclin B1
CHEK1	Checkpoint Kinase 1
CDK1	Cyclin Dependent Kinase 1
COL4A1	Collagen Alpha Chain 1
COL4A2	Collagen Type 4 Alpha Chain 2
COL6A1	Collagen Type 6 Alpha Chain 1
IL	Interleukin
TNF	Tumor Necrosis Factor
IFN	Interferon
MRLF	MRL/MpJ-Faslpr
PDT	Photodynamic Therapy
ALA	Aminolevulinic Acid
SP	Substance P
NEP	Neutral endopeptidase
NK1R	Neurokinin-1 receptor
NKA	Neurokinin A
NK-2R	Neurokinin-2 receptor
CRH	Corticotropin-Releasing Hormone
CGRP	Calcitonin gene-related peptide
NGF	Nerve Growth Factor
NPY	Neuropeptide Y
NO	Nitric Oxide
NOs	Nitric Oxide Synthase
GRP	Gastrin Releasing Peptide
VIP	Vasoactive Intestinal Peptide
PACAP	Pituitary Adenylate Cyclase-Activating Peptide
NT3	Neurotrophin-3
BDNF	Brain-Derived Neurotrophic Factor
PGP 9.5	Protein gene product 9.5
